# New Jersey Veterinary Medical Association

**Published:** 1892-05

**Authors:** W. H. Cooper

**Affiliations:** Secretary


### SOCIETY PROCEEDINGS.

*New Jersey Veterinary Medical Association.*—The New Jersey Veterinary Medical Association held its eighth annual meeting at the State Street House, Trenton, N. J., on Thursday, April 14th, 1892. The meeting was called to order at 11.30 A. M. by the President, Dr. R. R. Letts, of Hoboken, N. J. Secretary, Dr. W. H. Cooper, of Jersey City, called the roll, and the following Doctors answered to the call: Dr. J. W. Hawk, Newark, N. J.; Dr. J. C. Dustan, Morristown; Dr. B. F. King, Little Silver; Dr. W. B. E. Miller, Camden, Dr. John Kehoe, Lyndhurst; Dr. S. Lockwood, Woodbridge; Dr. W. Runge, Newark; Dr. W. H. Cooper, Jersey City; Dr. R. O. Hasbrouck, Passaic; Dr. R. R. Letts, Hoboken; Dr. J. Gerth, Jr., Newark; Dr. T. DeClyne, New Durham; Dr. J. M. Everitt, Hackettstown; Dr. M. M. Stage, Dover; Dr. A. W. Axford, Naughtright; Dr. H. Exton, Washington. Visitors: Dr. Ridge, of Pennsylvania; Dr. Sellers, of Camden; Franklin Dye, Secretary of State board of Agriculture of N. J.

A quorum being present, the minutes of the previous meeting were read and approved.

President, Dr. R. R. Letts, made his annual address.

The Secretary, Dr. W. H. Cooper, read his annual report, which was accepted, and ordered on file.

The Treasurer, Dr. B. F. King, read his annual report, which was accepted, and ordered on file.

The following applications for membership were received: Dr. J. N. Wittpenn, of Jersey City, N. J.; Dr. H. W. Elliott, of Newark, N. J.; Dr. Frank Lippincott, Swedesboro, N. J.; and Dr. B. L. Drummond, of Woodbridge, N. J.

Dr. Gerth moved a recess be taken for the Board of Censors to make their report. The chair appointed Drs. DeClyne, Dustan and Gerth to fill the vacancy in board.

The meeting was recalled to order. Dr. Hawk, chairman of the Board of Censors, made the following report. The board recommended for active membership Dr. H. W. Elliott, Graduate of N.Y. College, of Newark, N. J., Dr. B. L. Drummond, Graduate of American College, to this Association; Dr. J. N. Wittpenn, Graduate of the American College, and Dr. Frank Lippincott, to lay over until next meeting.

On motion the report was received and complied with.

The teller announced Drs Elliott and Drummond elected. The Chair introduced the newly elected members. The chair also introduced Dr. Ridge, of the Penn. Vet. Med. Association.

The following made reports: Dr. Dustan and Dr. Runge as delegates to the United States Association, by Dr. Miller, to the Pennsylvania Association.

No reports from the delegates to New York or Maryland Associations.

Unfinished business: Secretary made report of Dr. Davinson, of Asbury Park, motion to lay on table.

Reading of papers: Owing to the absence of Dr. Herbert, and sickness of Dr. Schuppan, the essayists for the meeting, a paper on "Colic" was read by Dr. B. F. King, Little Silver. A vote of thanks was given.

Miscellaneous business: Letters of regret were read. The letter of Dr. Smith was read and ordered on the table.

Motion to drop the names of Dr. Dimond and Dr. Wilkinson from the roll: ayes and nays being called, the motion was lost. Dr. Miller moved that the names of Dr. Dimond and Dr. Wilkinson be placed on the retired list; seconded and carried. Motion to take up delinquent members seconded and carried. Dr. Gerth moved that the Secretary and Treasurer go over the books, and meet the Board of Trustees, and make a report at the next meeting; seconded and carried.

The chair introduced the following gentlemen, who responded to the Association: Franklyn Dye, Secretary of the New Jersey State Board of Agriculture; Dr. Ridge, delegate from Pennsylvania State Veterinary Medical Association; Dr. A. T. Sellers, delegate from Keystone Veterinary Medical Association.

Discussion on Glanders by Drs. Hawk, Gerth, Runge and others; on Tuberculosis of the Gillingham herd, of Pennsylvania, by Drs. Miller, Dustan, Sellers, Ridge, Gerth and others.

Motion to proceed to the election of officers, seconded and carried; a motion by Dr. Gerth to appoint a committee of five, by the chair, to make nomination, was seconded and lost.

The President asked Dr. Miller to take the chair. Dr. Miller in the chair, asked for nomination for President, which was as follows: President, Dr. J. C. Dustan, Morristown; 1st Vice-President, Dr. W. Runge, Newark; 2nd Vice-President, Dr. S. Lockwood, Woodbridge; Secretary, Dr. W. H. Cooper, Jersey City; Treasurer, Dr. B. F. King, Little Silver. Board of Trustees: Chairman, Dr. W. B. E. Miller, Camden; Dr. J. W. Hawk, Newark; Dr. T. DeClyne, New Durham; Dr. R. O. Hasbrouck, Passaic; Dr. J. Kehoe, Lyndhurst.

Dr. Miller asked Dr. Letts to chair, who after taking the chair made an address to the Association and congratulated Dr. Dustan, and introduced him to the Association. Dr. Dustan, in taking the chair, made an address to the members.

The newly elected members were introduced. Adjournment for dinner.

The meeting was recalled to order by the President, Dr. Dustan; after a general debate and reporting of numerous cases, the chair made his appointments. Essayists for the next meeting: Drs. DeClyne, Schuppan, Gerth and Drummond. Delegates to the United States Veterinary Medical Association: Drs. Cooper, King, and Runge. Delegates to the New York Veterinary Medical Association: Drs. Gerth, Hasbrouck and DeClyne. Delegates to the Pennsylvania, Keystone and Maryland Veterinary Medical Associations: Drs. Miller, Cooper, Hawk and Letts. Place of next meeting at Newark, New Jersey, April 11th, 1892.

W. H. COOPER, *Secretary.*

